# Bacteraemia Caused by Probiotic Strains of *Lacticaseibacillus rhamnosus*—Case Studies Highlighting the Need for Careful Thought before Using Microbes for Health Benefits

**DOI:** 10.3390/pathogens11090977

**Published:** 2022-08-26

**Authors:** Agnieszka Mikucka, Aleksander Deptuła, Tomasz Bogiel, Agnieszka Chmielarczyk, Elżbieta Nurczyńska, Eugenia Gospodarek-Komkowska

**Affiliations:** 1 Department of Microbiology, Ludwik Rydygier Collegium Medicum in Bydgoszcz, Nicolaus Copernicus University in Torun, 85-094 Bydgoszcz, Poland; 2 Department of Propaedeutics of Medicine and Infection Prevention, Ludwik Rydygier Collegium Medicum in Bydgoszcz, Nicolaus Copernicus University in Torun, 85-094 Bydgoszcz, Poland; 3 Department of Microbiology, Faculty of Medicine, Jagiellonian University Medical College, Jagiellonian University, 31-121 Cracow, Poland; 4 Anesthesiology and Intensive Care Unit, University Hospital No 1 in Bydgoszcz, 85-094 Bydgoszcz, Poland

**Keywords:** bacteraemia, *Lacticaseibacillus* spp. infections, *Lacticaseibacillus rhamnosus*, microbiota, probiotics-related infections

## Abstract

Lactic acid bacteria belonging to *Lactobacillus* spp. and *Lacticaseibacillus* spp. are a natural part of fermented milk and other food products, probiotic supplements and human microbiota. They mainly belong to mucosal microflora, especially oral, vaginal and intestinal. *Lacticaseibacillus* spp. strains included in probiotics are generally characterised as safe microorganisms, and the species are concerned bacteria with very low pathogenic potential. However, infections caused by *Lactobacillus* spp. and *Lacticaseibacillus* spp., including bacteraemia and endocarditis, occur occasionally. The aim of the study was to present two cases of bacteraemia due to *Lacticaseibacillus rhamnosus* associated with the use of a probiotic product. It afflicted patients in intensive care units. The investigation was preliminarily based on clinical and microbiological recognition of the cases. The initial observation was laboratory confirmed with the application of pulsed-field gel electrophoresis (PFGE) results. Identical PFGE patterns were obtained for the evaluated strains and the strains derived from a commercially available probiotic that was administered to those patients. The increasing number of studies describing opportunistic infections due to probiotic strains of *Lacticaseibacillus* spp. should result in verifying the safety of probiotic formulations used in immunocompromised patients and forming detailed guidelines for the use of probiotics among patients from several risk groups.

## 1. Introduction

Lactic Acid Bacteria (LAB) belonging to *Lactobacillus* spp. and *Lacticaseibacillus* spp. are a Gram-positive component of human mucosal microflora, especially oral, vaginal and intestinal [1,2,3]. Some *Lactobacillus* spp. strains are used as probiotic bacteria in lyophilised formulations and fermented milk-based products. Their beneficial influence on both gut and vaginal microflora, also in the prevention of diarrhoea of different aetiology and *vaginitis* caused by *Candida* spp., was indicated; thus, these products are frequently used in outpatients and inpatients [4,5,6]. Interestingly, the role of *Lacticaseibacillus rhamnosus* (formerly *Lactobacillus rhamnosus*) GG strain is not limited to diarrhoea prevention. Additionally, it was indicated that this particular species is also involved in the primary prevention of eczema in children [7].

*Lactobacillus* spp., *Lacticaseibacillus* spp. and *Bifidobacterium* spp. representatives are characterised as bacteria with very low pathogenic potential, typical opportunistic pathogens, but it seems that it is not their properties but conditions favouring translocation that are crucial for bacteraemia originating from the gastrointestinal tract [2,8,9]. Thus, for many years, the rules of interpretation of LAB strains isolated from sterile sites have been debated. The opinions on the interpretation of *Lactobacillus* spp. and *Lacticaseibacillus* spp. isolation from the blood cultures are also discordant [1,10,11]. At the same time, an increasing number of reports on the isolation of these bacteria as aetiological factors of opportunistic infections are noted. The most frequently isolated species are *Lacticaseibacillus casei* and *L. rhamnosus* from blood cultures derived from patients with bacteraemia and *endocarditis* worldwide [1,10,11,12,13,14,15,16,17,18,19,20], including children and neonatal sepsis [21,22,23,24]. Some cases show organ/space infections due to LAB, such as *pneumonia, peritonitis, chorioamnionitis*, intraabdominal infections and abscesses [1,2,8,10,25,26,27,28]. Less frequently, these bacteria were isolated from patients with *pyelonephritis*, *endophthalmitis*, liver diseases, *meningitis* cases, infected wounds, leukaemia, transplantation and vascular grafts [1,8,29,30,31,32,33,34,35,36,37].

Most of the reported infections were classified as endogenous, but some of them were also reported as associated with the use of probiotic products containing *Lacticaseibacillus* spp. [1,11,14,38].

The most frequently described predisposing factors for LAB infections are immunosuppression associated with cancer, diabetes or immunosuppression in solid-organ transplant recipients. Another important risk factor for LAB bacteraemia is severe medical conditions requiring surgical intervention and long-term antimicrobial treatment with antimicrobials inactive against LAB [1,10,11,12,26,39].

This report describes two cases of bacteraemia due to *L. rhamnosus* in patients of the Department of Anaesthesiology and Intensive Care (ICU) and Clinical Unit of Anaesthesiology and Intensive Care with Cardiac Anaesthesiology Division (CICU) associated with the use of a probiotic product. The study was based on clinical and microbiological recognition of the infection cases. The observation results were laboratory confirmed with the application of pulsed-field gel electrophoresis (PFGE). The usefulness of molecular methods for the investigation of the source of bacteraemia, as well as for the analysis of *Lactobacillus* spp. and *Lacticaseibacillus* spp. strains relatedness was confirmed previously [14,40,41].

## 2. Clinical Cases Description

### 2.1. Case I Presentation

An 83-year-old male was admitted to the Intensive Care Unit (ICU) on 24 March 2011 with acute respiratory failure and haemorrhagic shock due to polytrauma. Haemopneumothorax was drained at Emergency Department, an open fracture of the right arm was stabilised with a triangular sling, and the wound was closed with a temporary suture. Final fracture stabilisation was delayed due to the unstable patient’s state, which was estimated at 21 in APACHE II and 18 on the SOFA scale, respectively. After complex diagnostics approach, the final diagnosis was established: acute respiratory failure, haemorrhagic shock, coagulopathy, multiple right side ribs fracture resulting in thorax instability, right side hemopneumothorax, right clavicle, scapula and humerus fractures, and compression fractures of the vertebral bodies at the levels Th3–Th6. Due to the continuous presence of blood in thorax drainage and progressive haemodynamic disorders, the decision to perform thoracotomy was made. After readmission to the ICU from the operation theatre, continuous venous–venous hemofiltration was introduced. After suspicion of ischaemia of the right arm, angiography and another CT of the thorax and abdomen were performed. The patient was qualified for the right arm fasciotomy and laparotomy due to persistent bleeding on the diaphragmal liver surface. Because haemostasis was impossible to achieve, packing was performed. After that, homeostasis was soon achieved, and the packaging was removed. On the fifth day after admission (28 March 2011), the metabolic state of the patient allowed the introduction of parenteral nutrition. Enteral nutrition was introduced on the following day. All the cultures were negative for 14 days (until 6 April 2011), and the patient’s condition was improving. On the 15th day (7 April 2011) patient’s state became worse again; in the physical examination, clinical signs of atelectasis, abdominal swelling with silent peristalsis and signs of infection in the right arm were revealed, and CRP level reached 400 mg/mL. From wound swabs and bronchoalveolar lavage (BAL) samples, *Pseudomonas aeruginosa* strain was cultured, which was sensitive to colistin and non-susceptible to carbapenems and fluoroquinolones. Colistin and meropenem were introduced in doses adjusted to CVVH (continuous venous–venous haemofiltration). In the following days, surgeons consulted the patient many times regarding abdominal pain and swelling. Abdominal CT revealed enlarged intestinal loops up to 7 cm; X-ray proved the presence of fluid in the intestines without signs of perforation. Because there were no indications for urgent laparotomy, prokinetics and probiotics were introduced. Loose stools occurred temporarily, and the patient tolerated enteral nutrition with a commercial solution. On the 35th day (27 April 2011), from five blood cultures *Lactobacillus* spp. strains were isolated, all sensitive to amoxicillin–clavulanate, which was introduced to the treatment. From BAL and wound swab samples, the *P. aeruginosa* strain sensitive only to colistin was isolated. The patient continued improving, but on the 46th day (8 May 2011), signs of abdominal sepsis occurred with a lactate level of 3.2. The patient was qualified for urgent laparotomy; during the surgery, partial resection of necrotic bowels was performed. In spite of intensive treatment, the patient died on the following day.

### 2.2. Case II Presentation

A 74-year-old female was admitted to the Department of Cardiac Surgery (29 November 2012) and eventually transferred to CICU (22 January 2013) due to acute respiratory failure two weeks after mitral valve replacement, tricuspid valve annuloplasty and coronary artery bypass grafting. The patient was intubated and ventilated mechanically. Due to suspected pneumonia, antimicrobial treatment with piperacillin/tazobactam was introduced. During hospitalisation, two strains of Gram-negative rods were isolated from the tracheal aspirate—*Stenotrophomonas maltophilia* and *Escherichia coli*—19 February 2013, and the patient received targeted antimicrobial treatment. From February 2013, the patient had persistent diarrhoea, but cultures for enteric pathogens (*Salmonella* spp., *Shigella* spp., *Yersinia* spp.) and the tests for *Clostridioides difficile* gave negative results. An ultrasound examination revealed no abnormalities in internal organs. On 2 February, 8 February and 17 February 2013, the patient received probiotics containing *Lactobacillus* spp.). On 21 February, 5 March, 8 March and 15 March 2013, symptoms of bacterial translocation from the gastrointestinal tract to the bloodstream were revealed—from several blood cultures, *Lactobacillus* spp. was isolated. Ampicillin was introduced for the treatment. After administration of ampicillin, there were no further *Lactobacillus* spp. Isolations, but other bacteria were cultured from BAL again (*S. maltophilia* and *E. coli* on 21 March, 31 March and 16 April 2013). Due to persistent diarrhoea, a colonoscopy was performed, which revealed unspecific colon inflammation, but the tests for *C. difficile* were negative again. On 4 April 2013, antimicrobial treatment was discontinued, with respiratory assistance remaining. In spite of intensive treatment, the patient’s state worsened, and on 19 April 2013, the patient died.

### 2.3. Laboratory Investigation

The laboratory part of the study involved 8 *Lacticaseibacillus* spp. strains obtained in a routine microbiology diagnostic procedure. Three of them (numbered 7838, 7894, 7914) were isolated from the first described patient in 2011, and five others (named 3708, 4804, 5035, 5039, 5447) were isolated from the CICU patient in 2013. Blood cultures were processed in Bactec (Becton–Dickinson, Franklin Lakes, NJ, USA) blood culture media; cultures were indicated as positive in the time between 25 h 14 min and 66 h 48 min. Gram stain revealed Gram-positive rods. The strains were cultured on Columbia Agar with 5% sheep blood (Becton Dickinson, Franklin Lakes, NJ, USA) at 37 °C supplemented with 5% CO_2_. The strain identification was performed with MALDI Biotyper (Bruker, Mannheim, Germany) according to the manufacturers’ instructions.

With the application of MALDI Biotyper (Bruker), all the strains were identified as *L. rhamnosus*, with an identification score of 2.035–2.280, category A— a reliable identification at the species level.

Antimicrobial susceptibility tests were performed according to Clinical and Laboratory Standards Institute: Methods for Antimicrobial Dilution and Disk Susceptibility Testing of Infrequently Isolated or Fastidious Bacteria, 2011 and 2013. All the E-tests with antibiotic gradients used for quantitative antimicrobial susceptibility evaluation were purchased from *bio*Mérieux.

All the strains were sensitive to: penicillin (MIC = 6 µg/mL), ampicillin (MIC = 6 µg/mL), gentamicin (MIC = 1 µg/mL), erythromycin (MIC = 0.094 µg/mL), clindamycin (MIC = 0.008 µg/mL), while resistant to: cefotaxime (MIC > 32 µg/mL), imipenem (MIC > 32 µg/mL), and vancomycin (MIC > 256 µg /mL). MIC value for ciprofloxacin was 3 µg/mL, and for levofloxacin, 0.75 µg/mL, but there were no interpretation criteria for fluoroquinolones according to CLSI.

Because both patients obtained probiotic agents, the strains isolated from patients were compared with the application of molecular methods with the strains included in two probiotic agents—Lacidofil (Lallemand SAS, Blagnac, France) and Dicoflor (Bayer, Leverkusen, Germany).

The identity of the isolated *L. rhamnosus* strains was compared using the pulsed-field gel electrophoresis technique. Briefly, genomic DNA was isolated from bacteria plunged in agarose blocks by lysis with a lytic buffer supplemented with lysozyme (2.5 mg/mL; Sigma, Watford, UK) and mutanolysin (20 U/mL; Sigma, Watford, UK). Then, the blocks were incubated for 18 h at 50 °C with proteinase K (1 mg/mL, Fermentas, Waltham, MA, USA) and digested with *Sgs*I restriction nuclease (25 U/plug, Fermentas, Waltham, MA, USA) overnight at 37 °C. Electrophoresis was carried out with a CHEF III apparatus (Bio-Rad) in 1% certified agarose (Bio-Rad, Hercules, CA, USA) with a 0.5× TBE buffer (Bio-Rad, Hercules, CA, USA). The pulse time was 1–25 s, the current was 5.5 V/cm, the temperature was 14°C and the running time was 24 h. The gel was stained with ethidium bromide and analysed using GelCompar (Applied Maths, Sint-Martens-Latem, Belgium).

*L. rhamnosus* strains isolated from 5 blood cultures collected between 17 February 2013 and 10 March 2013 from the CICU patient (case II) were identical to *L. rhamnosus* strains isolated from the ICU patient in 2011, and all these strains were identical with *L. rhamnosus* strain derived from a probiotic product Lacidofil (Supplementary Materials Figure S1).

## 3. Discussion and Conclusions

Despite an increasing number of reports describing bacteraemia episodes due to LAB within the last 20 years, and despite its authors emphasising its important role in opportunistic infections, there are no guidelines for diagnostics of infections due to LAB [1,5,10,11,12,26,38,42]. As was previously indicated by other authors, the interpretation of the results is not only difficult but the culture and identification of these bacteria is also quite a problematic issue in each microbiology laboratory [42]. These bacteria require enriched culture media and a capnophilic or anaerobic atmosphere; sometimes, culture time on solid media should be extended up to 48 h. Identification of these bacteria with biochemical methods, which are available in most microbiology laboratories, is impossible or ambiguous. Hence, detailed phenotypic or molecular (e.g., 16S rRNA sequencing) or mass spectrometry (MALDI-TOF MS) methods should be involved, but they are not widely available [17,43,44,45]. In both described cases, growth of bacteria was observed after 48 h, and the final identification of the strains was possible only with MALDI-TOF MS.

Moreover, for the differentiation of *Lactobacillus* spp. and *Lacticaseibacillus* spp. strains and the evaluation of their relatedness sequencing or PFGE techniques can be applied since their usefulness was confirmed previously, also PFGE with the application of *Sgs*I restriction enzyme [14,40,41].

There are no European guidelines for antimicrobial susceptibility testing for *Lactobacillus* spp., so CLSI standards should be used, or the results should be interpreted according to EUCAST breakpoints for non-related species. In situations as described above, when LAB strains are isolated from several blood cultures, there are no doubts that they should be classified as an aetiological factor of bacteraemia, but authors are inconsistent in the case of single positive specimens [1,4,10,11,12,26,38]. Some of the authors believe that in that cases, LAB should be treated as contaminants, despite the fact that they are not members of skin microbiota and their role in catheter-related bloodstream infection was not documented [10,12,26].

Nevertheless, it seems to be justified to propose constant and reliable criteria for interpretation of positive blood cultures revealing *Lactobacillus* spp. growth. Husni et al. [12] divided cases of isolation of *Lactobacillus* spp. for definite (at least two positive blood cultures or one positive blood culture and deep-site-derived specimen), probable (one positive blood culture in case of mixed bacteraemia—co-culture with another microorganism classified as clinically significant) and possible (one positive blood culture with signs and symptoms of an infection referable to a site likely to yield to the organism).

At the same time, many authors indicate that there are several risk factors for bacteraemia due to LAB, such as severe underlying disease (cancer, transplant recipients, diabetes), prolonged hospitalisation and antimicrobial treatment with drugs inactive against LAB, such as vancomycin or third-generation cephalosporins [1,4,10,11,12,26,38]. Other important factors predisposing for LAB translocation from the gastrointestinal tract are selective digestive tract decontamination and abdominal surgery, including endoscopic procedures [10,12,26]. In the case of patients with particular risk factors, *Lactobacillus* spp., *Lacticaseibacillus* spp., *Lactiplanibacillus plantarum*, *Limosilactobacillus fermentum* as well as other genera and species should be considered as a potential aetiological factor, and appropriate antimicrobial treatment should be introduced [12,42,46].

*Lacticaseibacillus* spp. infections might be not only endogenous but also exogenous, related to the use of probiotic formulations [11,14,38], which are similar to those presented cases. Because consumption of probiotic food is increasing and there is no international consensus on methodology for testing the efficacy and safety of probiotics [4,38], the Food and Agriculture Organization of the United Nations (FAO) and World Health Organization (WHO) started work on the evaluation of scientific data on safety and functional aspects of probiotics [5]. This work resulted in the recommendation that microorganisms, which are used as probiotics, have to present a history of safe use and have to be tested for pathogenicity, toxigenicity and virulence. FAO/WHO experts recommend that also side effects should be evaluated during clinical trials as well as lack of infectiveness in immunocompromised animals should be proven [5]. However, in the case of patients from several risk factors groups (e.g., severe underlying conditions, immunosuppression, undergoing surgical procedures, during antimicrobial treatment), special recommendations on the safety of probiotics use should be elaborated because the reported incidence of infections due to probiotic strains is between 1.7 and 8% [1,6,11,36,38].

Both bacteria and extracellular factors produced by them affect the intestinal barrier, but factors such as mucosal damage, immunosuppression, abnormal changes in the intestinal microbiome and visceral ischaemia, which may accompany many underlying conditions, play a crucial role. It is difficult to induce translocation in healthy individuals, and because of that, the pathomechanism of this process is still studied [4,9]. In light of metagenomic studies and the concept of the superorganism, where functioning is based on cooperation between microbiota and the human body, explaining the translocation process might be much more difficult than expected.

Problematic issues are also signs and symptoms, as well as the mortality in infections due to LAB. Bacteraemia might be asymptomatic or have very unspecific signs limited to fever and leucocytosis, but it also may lead to sepsis or developing other complications such as endocarditis or intraabdominal abscesses, including among healthy people [1,8,10,11,12,13,25,26]. Mortality among patients with LAB infections is estimated at the level of 30%, but usually, the direct causes of death are underlying conditions or accompanying infections [1].

In both presented cases, patients died in spite of an introduction of antimicrobials, which were active against *L. rhamnosus*, and the direct death causes were rather underlying conditions, not an infection itself.

An introduction of antimicrobials active against *Lactobacillus* spp. is also of great importance, but Cannon et al. [1] proved that the differences in the death rates were statistically insignificant. Some of the authors of studies on the clinical importance of *Lactobacillus* spp. claimed that bacteraemia due to these microorganisms might be the marker of severe underlying diseases as well as poor overall prognosis [11,12,26]. According to Salminen et al. [42], the survival of patients after *Lactobacillus* spp. bacteraemia is 88%, 74% and 52%, respectively, within one week, one month and one year.

That is why it is also recommended to introduce antimicrobial treatment active against *Lactobacillus* spp. for immunocompromised patients [11,42]. Studies from the beginning of this century prove that penicillin, ampicillin and aminoglycosides are active against *Lactobacillus* spp. and *Lacticaseibacillus* spp., and over 90% of strains are also sensitive to erythromycin and clindamycin. However, since lincosamides and macrolides are bacteriostatic, they are not recommended for the treatment of life-threatening infections. The activity of fluoroquinolones and cephalosporins is variable, but usually, second-generation cephalosporins are more active than the third. In line with the CLSI recommendations, antimicrobial susceptibility testing interpretation criteria for LAB are only available for penicillin, ampicillin and carbapenems (imipenem and meropenem). Since at least 2015, there have been no such criteria for aminoglycosides. However, some researchers recommend combined therapy (penicillin, ampicillin, and aminoglycoside), with new fluoroquinolones being considered as alternative options. Therefore, the sensitivity of the strains to the aforementioned antimicrobial agents was investigated [1]. The lowest percentage of sensitive strains is reported for vancomycin, but there are some species, which might be sensitive to that antimicrobial, i.e., *L. acidophilus*, *L. jensenii*, *L. gasseri*, so the species-level identification of *Lactobacillus* spp. or *Lacticaseibacillus* spp. play an important role [1,39,42].

It is well known that acidification of the environment caused by metabolites synthesised by LAB at the site of infection may reduce the activity of aminoglycosides and penicillins (it reduces the bactericidal activity of beta-lactams), regardless of the actual location of an infection. Therefore, higher doses of penicillins or their combination with aminoglycosides are recommended for the treatment [1,42]. Some of the authors recommend that treatment regimen only in case of endocarditis or deep-site infections [12,26]. In both of the presented cases, the *L. rhamnosus* strain was resistant to cephalosporins, imipenem and vancomycin, but the applied antimicrobial treatment with aminopenicillins should be effective in the treatment of bacteraemia.

The increasing number of studies describing opportunistic infections due to probiotic strains of *Lacticaseibacillus* spp. [46] should result in verifying the safety of the use of probiotic formulations in immunocompromised patients and forming the guidelines for the use of probiotics in patients from several risk groups.

## Data Availability

The data presented in this study are not publicly available as a matter of confidentiality. However, these data are available upon request from the corresponding author.

## Supplementary Materials

The following supporting information can be downloaded at: https://www.mdpi.com/article/10.3390/pathogens11090977/s1, Figure S1. PFGE typing profiles of *L. rhamnosus* obtained with *Sgs*I restriction enzyme digestion.
