# Supplementary material for: Bacteraemia Caused by Probiotic Strains of Lacticaseibacillus rhamnosus—Case Studies Highlighting the Need for Careful Thought before Using Microbes for Health Benefits
Source: Pathogens. 2022 Aug 26;11(9):977. doi: 10.3390/pathogens11090977 (PMC9504050; doi:10.3390/pathogens11090977)
Supplement: Supplementary file 1 [file pathogens-11-00977-s001.zip › pathogens-1854276-supplementary.pdf]

Bacteraemia caused by probiotic strains of *Lacticaseibacillus rhamnosus* – case studies highlighting the need for careful thought before using microbes for health benefits

Agnieszka Mikucka[1], Aleksander Deptuła[2], Tomasz Bogiel[1*], Agnieszka Chmielarczyk[3], Elżbieta Nurczyńska[4], Eugenia Gospodarek-Komkowska[1]

[1] Department of Microbiology Ludwik Rydygier Collegium Medicum in Bydgoszcz, Nicolaus Copernicus University in Torun, Poland

[2] Department of Propaedeutics of Medicine and Infection Prevention Ludwik Rydygier Collegium Medicum in Bydgoszcz, Nicolaus Copernicus University in Torun, Poland

[3] Chair of Microbiology, Jagiellonian University Medical College in Cracow, Poland

[4] Anesthesiology and Intensive Care Unit, University Hospital No 1 in Bydgoszcz, Poland

*** Correspondence:** 9 Maria Skłodowska-Curie Street, 85-094 Bydgoszcz, e-mail: t.bogiel@cm.umk.pl (T.B.), telephone number: +48 52 585 44 80

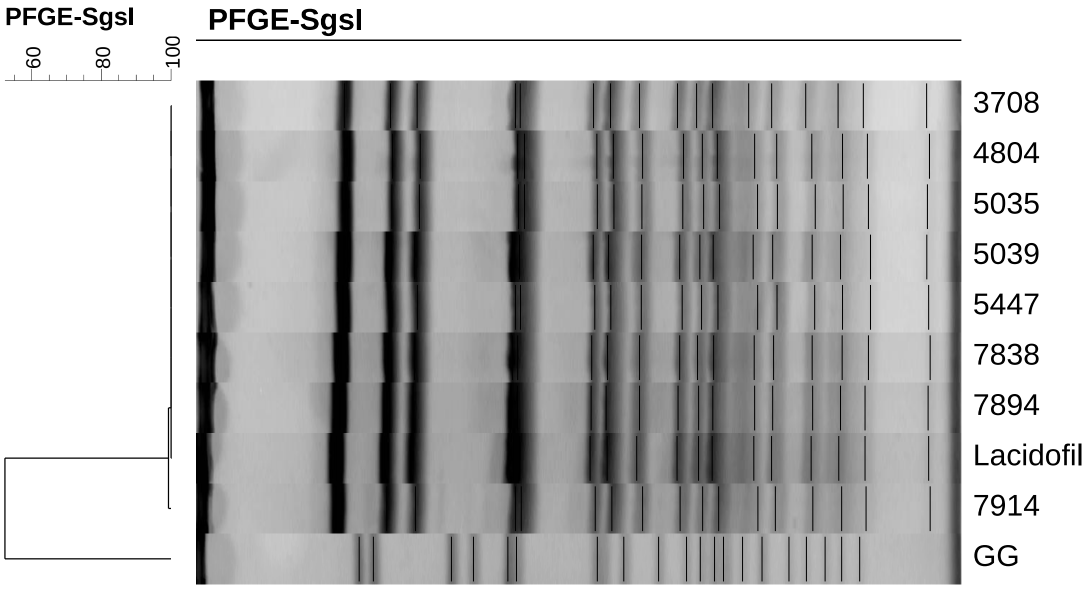

Figure S1. PFGE typing profiles of *L. rhamnosus* obtained with *Sgs*I restriction enzyme digestion. Analysis was done in Gel Compar software (coefficient Dice, UPGMA algorithm, UPGMA, Tol. 1%, Opt 0.5%, Min area 0.0%), 3708-7914 – the strains isolated from the patients, Lacidofil – probiotic strain *L. rhamnosus* derived from Lacidofil (LALLEMAND SAS); GG – *L. rhamnosus* strain GG isolated from Dicoflor (Bayer)
